# A prediction method for radiation proctitis based on SAM-Med2D model

**DOI:** 10.1038/s41598-025-87409-6

**Published:** 2025-04-18

**Authors:** Ning Zhang, Haifeng Ling, Wenyu Zhang, Mei Zhang

**Affiliations:** 1 Department of Radiotherapy, The First Affiliated Hospital of Anhui Medical University, Hefei, 230000 China; 2 School of Computer Science and Technology, University of Science and Technology of China, Hefei, 230000 China; 3 Oncology Department of Integrated Traditional Chinese and Western Medicine, The First Affiliated Hospital of Anhui Medical University, Hefei, 230000 China; 4 Graduate School of Anhui University of Chinese Medicine, Hefei, 230000 China; 5 The Traditional and Western Medicine (TCM)-Integrated Cancer Center of Anhui Medical University, Hefei, 230000 China

**Keywords:** Computational models, Image processing, Machine learning, Radiotherapy

## Abstract

Cervical cancer, a prevalent gynecological malignancy, poses significant threats to women’s health. Despite advances in treatment modalities, radiotherapy remains a cornerstone in managing cervical cancer. However, radiotherapy-induced complications, such as radiation proctitis, present substantial diagnostic and prognostic challenges. Accurate diagnosis are crucial for optimizing treatment strategies and improving patient outcomes. Deep learning has shown remarkable success in medical image segmentation, aiding clinicians in assessing patient conditions. In the other hand, radiomics excels in extracting diagnostically valuable features from medical images but requires extensive manual annotation and often lacks generalizability. Therefore, combining the strengths of deep learning and radiomics is pivotal in addressing these challenges. In this study, we propose a novel paradigm that leverages deep learning models for initial segmentation, followed by detailed radiomics analysis. Specifically, we utilize the Transformer-based SAM-Med2D model to extract visual features from CT images of cervical cancer patients. We apply T-tests and Lasso regression to identify features most correlated with radiation proctitis and build predictive models using logistic regression, random forest, and naive Gaussian Bayesian algorithms. Experimental results demonstrate that our method effectively extracts CT imaging features and exhibits excellent performance in diagnosis radiation proctitis. This approach not only enhances predictive accuracy but also provides a valuable tool for personalizing treatment plans and improving patient outcomes in cervical cancer radiotherapy.

## Introduction

Cervical cancer, also known as carcinoma of the cervix, is a common gynecological malignancy occurring in the cervix, posing a significant threat to women’s health. Common treatment methods for cervical cancer include surgery, radiotherapy, chemotherapy, targeted therapy, and immunotherapy. Among these, radiotherapy uses high-energy rays to kill or inhibit the growth of cancer cells. External beam radiotherapy (EBRT) and brachytherapy are the primary radiotherapy techniques for treating cervical cancer. EBRT involves mapping the radiation target areas by radiation oncologists and irradiating the tumor and metastatic sites multiple times over a specified period. Brachytherapy, on the other hand, involves placing a radioactive source near or within the tumor using specialized catheters. Increasing the radiation dose to the target area during radiotherapy can enhance local control rates of cervical cancer and improve efficacy. However, high-dose radiation also increases the radiation toxicity to organs such as the rectum, sigmoid colon, bladder, and vagina, thereby increasing the risk of radiotherapy-related complications.

Radiation proctitis is an intestinal complication induced by radiotherapy for pelvic, abdominal, and retroperitoneal malignancies, potentially affecting the small intestine, colon, and rectum, hence also referred to as radiation proctitis, colitis, or proctitis. Depending on the radiation dose, duration, and onset, radiation proctitis is generally classified into acute and chronic forms. In the early stages, radiation inhibits the renewal of intestinal mucosal cells, causing swelling and occlusion of small arteries, leading to ischemia and mucosal erosion of the intestinal wall. In later stages, fibrosis, intestinal stenosis, gastric perforation, intra-abdominal abscesses, fistulas, and adhesions may occur. Early diagnosis of the condition allows for the determination of optimal treatment plans, increasing the patients’ survival probability.

Medical imaging modalities such as ultrasound, X-ray, CT, MRI, and PET not only facilitate convenient detection but also directly visualize the organs and their lesions, as well as their invasion into adjacent or distant organs, offering unique predictive value. Medical imaging contains rich information, requiring experienced clinicians to spend significant effort to obtain or interpret it, such as manually identifying and segmenting lesion areas. In these scenarios, computerized methods can enhance the predictive process by providing supplementary interpretable data from multimodal imaging and aiding in the structured interpretation of standard radiologic images, thereby improving prediction accuracy^1,2^. Traditional methods involve extracting handcrafted radiomic features based on specific formulas and definitions^3–5^, leveraging the biological significance of these features and their semantic relationships in describing lesions to assist in clinical diagnosis and prognosis prediction. Imaging omics methods have achieved success in tasks such as predicting radiation toxicity and have been found to be effective in predicting radiotherapy outcomes and disease-free survival^6–8^. These methods provide a non-invasive and quantitative evaluation for exploring the potential depth information of diseases, and have been applied in predicting acute/late toxicity after radiotherapy for various cancers^9^. While these methods have demonstrated success in predicting radiation toxicity and radiotherapy outcomes, their clinical application faces substantial limitations^10^. The manual feature extraction process demands considerable expertise and time investment from clinicians, introducing potential inter-observer variability^11^. Moreover, the rigid nature of predefined formulas often fails to capture subtle biological characteristics that may be clinically significant^12^. The generalizability of these handcrafted features remains particularly challenging, as their effectiveness can vary significantly across different imaging protocols and institutions^13^. These limitations have increasingly motivated the exploration of more advanced computational approaches.

The evolution from predefined manual features to deep learning features in radiomics has been spurred by advances in artificial intelligence and computing devices, with deep learning-based radiomics demonstrating significant results across various fields^14–16^. This, deep learning neural networks like convolutional neural networks (CNNs) and their variants have also shown excellent performance in medical image segmentation and recognition tasks^17–19^. The Transformer architecture, initially used in natural language processing, has been adapted to vision tasks such as image classification and object detection, and has excelled in processing medical images and tasks such as medical image classification and disease diagnosis^20,21^. Recent studies have showcased the effectiveness of the Transformer architecture in numerous medical applications, including diagnosing COVID-19 from X-ray images, classifying skin cancer, and detecting abnormalities in chest X-ray images^22–25^. The adaptation of the Transformer architecture to computer vision has led to remarkable results in various medical tasks, such as predicting the survival rate of rectal cancer patients using the Vision Transformer to extract features from MRI data^26^, and the development of highly performant and generalizable medical image segmentation models like MedSAM^27^ and SAM-Med2D^28^. However, the application of deep learning in diagnosis prediction presents unique challenges. One major concern is the ’black box’ nature of these models, which poses significant issues in clinical settings where interpretability is crucial for decision-making^29^. Additionally, deep learning models require large amounts of labeled training data, a resource that is often scarce in medical contexts. Furthermore, the features automatically learned by these models may not always correspond to clinically relevant characteristics, potentially limiting their practical utility in diagnosis prediction^11^.

Deep learning has demonstrated exceptional performance in image segmentation, effectively assisting doctors in evaluating patient conditions. However, its performance in prognostic prediction remains suboptimal. On the other hand, radiomics plays a crucial role in medical diagnosis and prognosis prediction by extracting highly diagnostic imaging features^30^. Nonetheless, it requires manual annotation of regions of interest by doctors and often lacks generalizability. How to precisely locate diseased areas in patients to assist doctors in diagnosis and evaluation, and provide reliable prognostic predictions, is a significant challenge.

We propose a novel paradigm that integrates deep learning and radiomics to predict the likelihood of radiation proctitis in cervical cancer patients undergoing radiotherapy. Our approach leverages the SAM-Med2D model, a Transformer-based architecture, to automatically extract visual features from CT images. With the help of SAM-Med2D, we achieve more accurate and representative feature extraction compared to traditional radiomics methods. We select features correlated with radiation proctitis using statistical methods and build predictive models using logistic regression, random forest, and naive Gaussian Bayesian algorithms. These models demonstrate acceptable performance with the logistic regression model achieving the best performance of all models considered, as measured by an AUC value of around 0.73, providing valuable references for clinical decision-making in treatment planning. Additionally, our method reduces the manual annotation burden on clinicians while maintaining clinical interpretability, making it highly suitable for widespread clinical adoption. Our contributions are as follows:

1. *Novel Deep Learning-Radiomics Framework*: We developed an innovative framework integrating SAM-Med2D with radiomics analysis, bridging the gap between deep learning’s feature extraction capabilities and clinical interpretability for identifying patients at risk of radiation proctitis;
2. *Robust Feature Selection and Model Development*: We implemented a systematic pipeline using statistical analysis and Lasso regression for feature selection, ensuring both statistical reliability and clinical relevance. The models predict the risk of radiation proctitis with an AUC of around 0.73;
3. *Enhanced Clinical Applicability and Interpretability*: Our framework reduces manual annotation burdens and provides anatomically interpretable predictions, aiding in diagnostic decision-making and making it highly suitable for clinical adoption in radiotherapy planning.

## Methods

### Research subjects

We conducted a retrospective study on cervical cancer patients who received radical radiotherapy at our hospital between June 2017 and January 2022. The criteria for selecting patients for inclusion are as follows: 1 pathological diagnosis of cervical squamous carcinoma stage IIA-IIIB; 2 radical radiotherapy; 3 age 18-75 years. The exclusion criteria are as follows: 1 incomplete clinical data; 2 combined with inflammatory bowel disease or other causes of colitis; 3 undergoing immunotherapy prior to, during, and for a period of 6 months following radiotherapy; 4 experiencing a second primary tumor or a systemic chronic wasting disease; 5 visual distortions caused by imaging techniques. Following a thorough screening and exclusion process, a total of 120 patients were ultimately selected to participate in the study. This study was conducted in accordance with the Declaration of Helsinki, and approved by the Clinical Research Ethics Committee of the First Affiliated Hospital of Anhui Medical University (Ethical Review-Quick-PJ2024-04-73). Written informed consent was obtained from all the patients prior to this study.

### Image acquisition

Prior to imaging acquisition, neither surgery nor simultaneous radiotherapy was conducted. Pelvic CT scan was conducted using the CT GE Discovery CT590 RT scanner prior to the commencement of treatment. The bladder was maintained at approximately full capacity during the CT examination. The radiotherapy was conducted using the Eclipse treatment planning system V15.6 (Varian Medical Systems, Palo Alto, CA) under the supervision of skilled physicians and physicists. The original CT images were stored in a picture archiving and communication system (PACS) and then converted to DICOM format using the sequences stored in PACS. The data to be analyzed consists of CT images from 150 cervical cancer patients, collected before they underwent radiation therapy.

### Pipeline of method

**Fig. 1:**
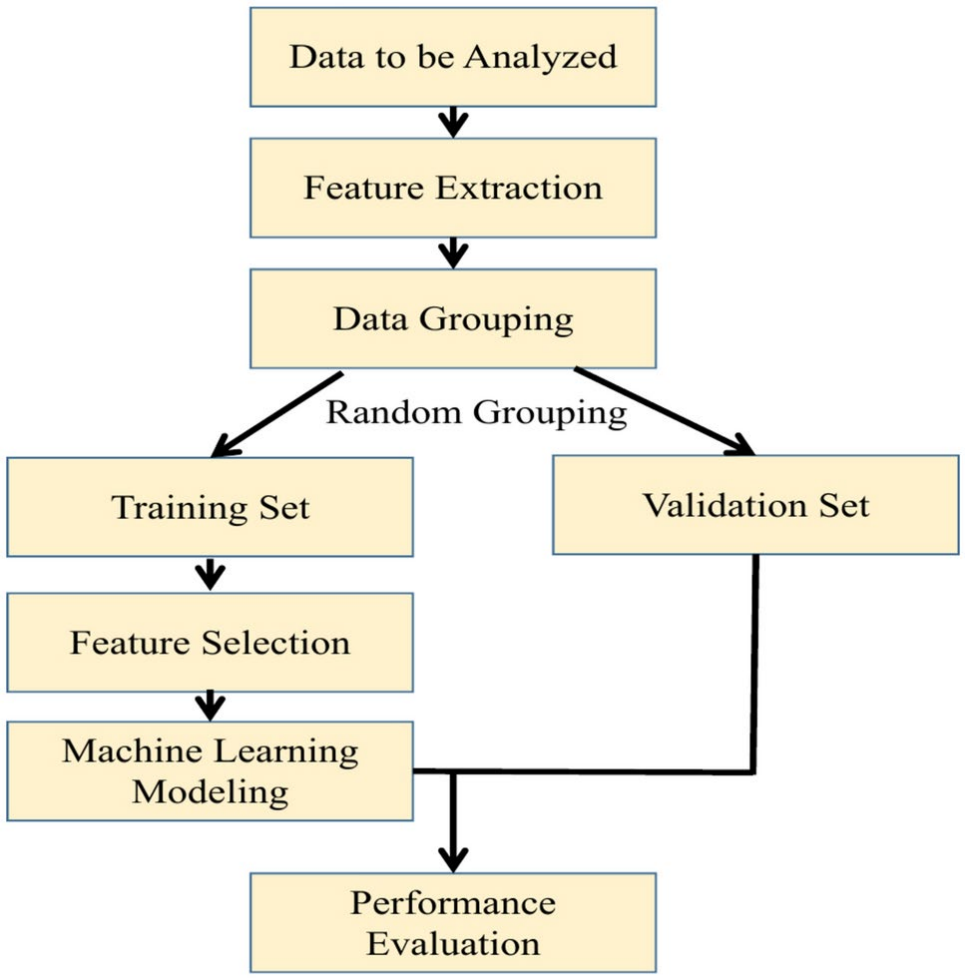
Data analysis flow.

This study aims to explore the application of Transformer-based deep learning methods in medical imaging analysis, specifically in extracting imaging features to predict the likelihood of patients developing radiation proctitis following radiotherapy. As illustrated in Fig. 1, upon acquiring the data to be analyzed, we utilized the state-of-the-art Transformer model, SAM-Med2D^28^, to obtain the regions of interest (ROI). Through its powerful self-attention mechanism, SAM-Med2D effectively captures spatial and contextual information within the images. To ensure the accuracy and comprehensiveness of feature extraction, we selected the slice with the largest cross-sectional area of the rectum. Subsequently, we employed T-tests and LASSO regression for feature selection, and logistic regression, random forest, and Gaussian naive Bayes to construct predictive models. We evaluated the robustness and generalization ability of the models using 5-fold cross-validation. By combining various evaluation metrics, including accuracy, precision, recall, specificity, F1 score, and AUC value, we comprehensively assessed the performance of the models. The results of this study demonstrate the potential of deep learning methods in medical imaging analysis, providing strong support for risk prediction in clinical radiotherapy.

### Overview of the SAM-Med2D model

**Fig. 2:**
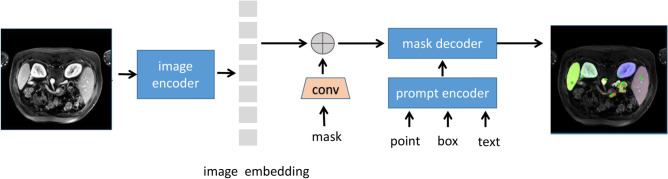
The structure of the SAM-Med2D model fine-tunes the prompt encoder using point, Bbox, and mask information and updates the mask decoder’s parameters through interactive training.

The SAM-Med2D is a fine-tuned version of the segmentation model SAM^31^ specifically adapted for the medical imaging domain, effectively extending its capabilities to this field. As illustrated in Fig. 2, the SAM-Med2D model comprises an image encoder, a prompt encoder, and a mask decoder. Leveraging the powerful capabilities of SAM, it effectively captures spatial and contextual information within the images, extracting high-dimensional, high-quality features. These features not only encompass local image details but also retain global structural information, thereby demonstrating excellent performance in analysis and prediction tasks.

**Fig. 3:**
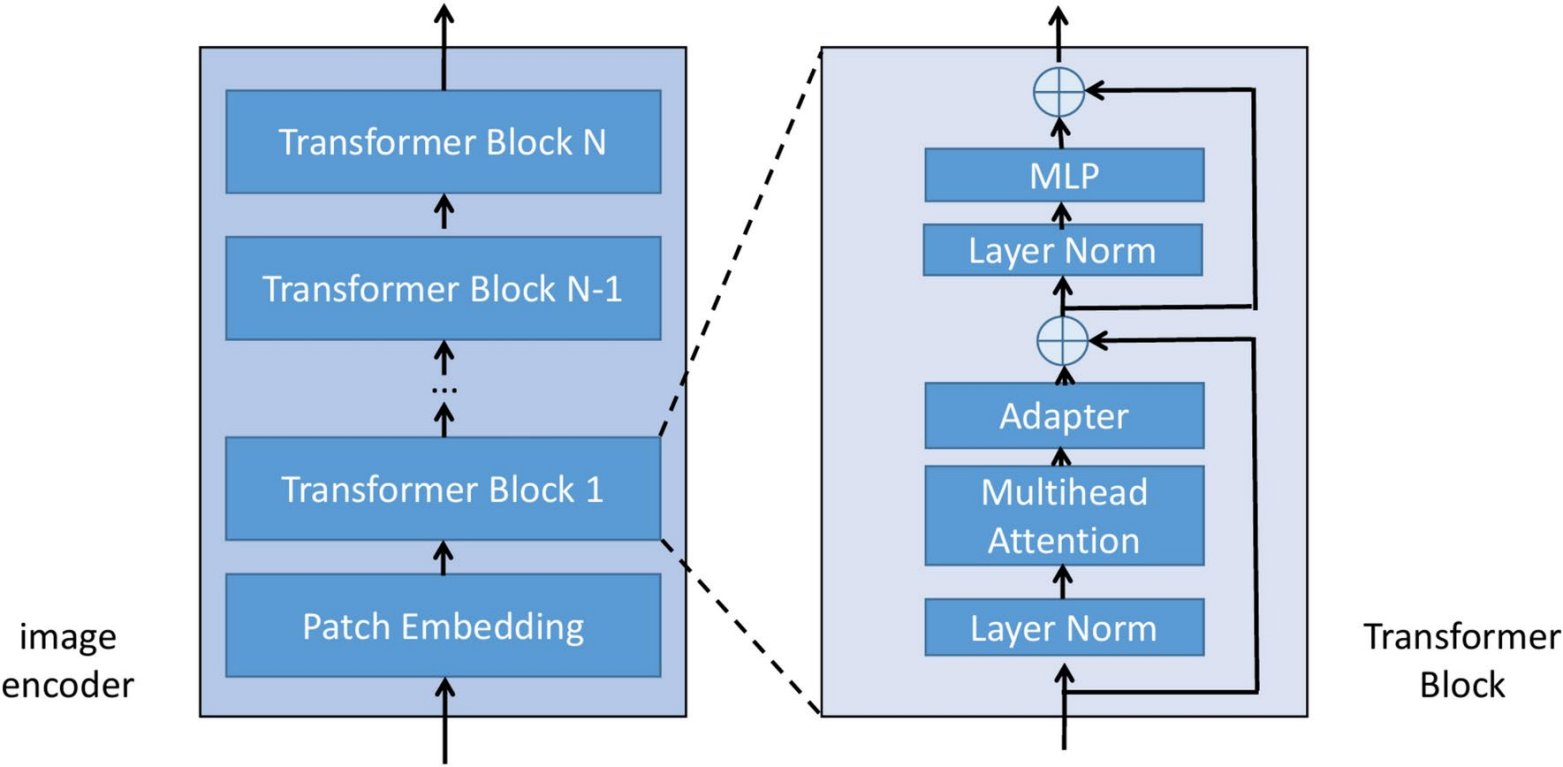
In the SAM-Med2D model, the image encoder is frozen, and learnable adapter layers are incorporated into each Transformer block to acquire domain-specific knowledge in the medical field.

The image encoder of SAM-Med2D is the Vision Transformer (ViT), as illustrated in Fig. 3. ViT is an innovative neural network architecture that divides images into a series of small patches and uses a self-attention mechanism to capture the complex relationships between these patches. The features obtained from these patches support image analysis tasks. The core structure of the Vision Transformer consists of Transformer blocks, with SAM-Med2D comprising 12 Transformer blocks. The fundamental idea behind the Transformer block is the attention mechanism, where the input is first multiplied by the respective matrices $W^Q$, $W^K$, and $W^V$ to derive the query matrix $Q$, key matrix $K$, and value matrix $V$. Subsequently, the attention matrix $Z$ is computed using the softmax function. The calculation is given by the formula below, where $d_k$ represents the dimensionality of the key matrix $K$:

1 $$\begin{aligned} Z = \text {ATTN}(Q, K, V) = \text {softmax}\left( \frac{Q \cdot K^T}{\sqrt{d_k}} \right) V, \end{aligned}$$

Within the Transformer block, a multi-head attention mechanism (MSA) is utilized. This mechanism involves computing multiple attention matrices, concatenating these attention matrices, and then multiplying the concatenated result by a new weight matrix $W_0$ to obtain the final attention matrix. The formula is expressed as follows:

2 $$\begin{aligned} \text {MSA} = \text {Concat}(Z_1, Z_2,..., Z_h) W_0, \end{aligned}$$

The SAM-Med2D model first normalizes and resizes the images, after which the image encoder of the model begins its work. Starting with Patch Embedding, a specially designed convolutional layer is used to divide the image into patches $[x_1^pE; x_2^pE; ...; x_n^pE]$, Where ${E}$ is a linear transformation that converts each patch into a depth feature vector, ultimately resulting in $16 \times 16$ vectors of 256 dimensions. Positional encoding $E_{pos}$ is then added to provide the model with positional information of each pixel or patch in the image, enabling the model to understand the spatial arrangement of objects within the image for more accurate image segmentation tasks. The specific computation formulas for the Vision Transformer are shown in equations 3 to 7:

3 $$\begin{aligned} z_0 = \left[ x_1^pE; x_2^pE;...; x_n^pE \right] + E_{pos}, \quad E \in \mathbb {R}^{(P^2 \cdot C) \times D}, \quad E_{pos} \in \mathbb {R}^{N \times D}, \end{aligned}$$

4 $$\begin{aligned} z'_l = \text {MSA}(\text {LN}(z_{l-1})) + z_{l-1}, \quad l = 1,..., L, \end{aligned}$$

5 $$\begin{aligned} z''_l = \text {Adapter}(z'_l), \quad l = 1,..., L, \end{aligned}$$

6 $$\begin{aligned} z_l = \text {MLP}(\text {LN}(z''_l)) + z''_l, \quad l = 1,..., L, \end{aligned}$$

7 $$\begin{aligned} y = \text {LN}(z_L), \end{aligned}$$

where $P$ is the patch size; $C$ is the number of channels; $D = 256$ is the dimension size; $N = 16 \times 16$; and $L$ is the number of Transformer layers, which equals 12. MSA denotes multi-head attention mechanism; Adapter represents a network structure for fine-tuning the SAM model, consisting of fully connected layers; LN stands for layer normalization; and MLP refers to the multi-layer perception.

The prompt encoder allows for the direct indication of regions of interest on the original image to guide the model. These prompt signals are converted into features that match the spatial embedding of the image through convolution operations, and then combined with the image embeddings to provide precise positional information for segmentation. Prompts can be in the form of points or boxes; a point is a user-drawn spot on the original image, and after processing by the prompt encoder, it has a size of $1 \times 256$. Similarly, $N$ points form $N \times 256$. Points are labeled as positive or negative, where positive indicates foreground and negative indicates background. Therefore, a set of learnable parameters representing the embeddings of positive and negative points (denoted as point_embedding, $1 \times 256$) is added to the $1 \times 256$ feature of each point.

**Fig. 4:**
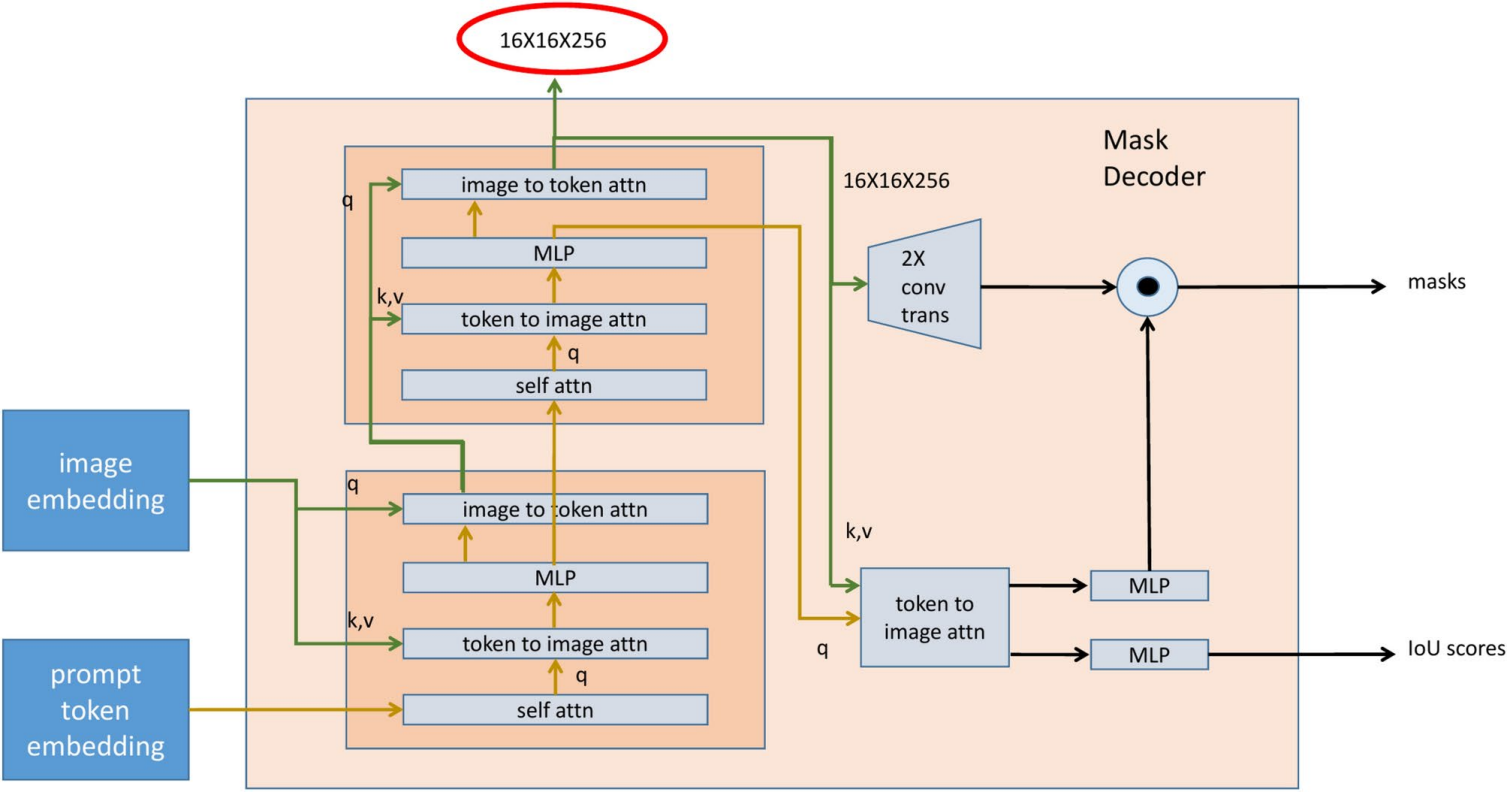
Mask decoder block diagram.

The mask decoder is a structure designed to obtain segmentation results, as illustrated in Fig. 4. Initially, for the output tokens generated by the prompt encoder, the mask decoder first applies a self-attention mechanism. The role of self-attention is to enable the model to search for and amplify important relationships within the tokens, which is crucial for understanding the interactions between various prompt information. The computation follows the same formula as Equation 1, where the query matrix $Q$, key matrix $K$, and value matrix $V$ are all derived from the same input $x$.

Next, these tokens, processed by the self-attention mechanism, serve as queries and are combined with the keys and values obtained from the image embeddings output by the image encoder, performing an image-to-token attention operation. This step aims to integrate the information processed by the prompts with the rich contextual information of the original image, thereby introducing visual information into the decoding process. The result is then passed through a multi-layer perceptron (MLP) layer, generating new feature representations. These feature representations are subsequently used as keys and values, with the image embeddings serving as queries, to perform another round of image-to-token attention, further refining the model’s understanding of the image. This process constitutes the first part of the mask decoder.

The second part employs the same structure to further enhance the feature representations. In the output stage of the model, there are two branches. One branch maps the features back to the original image size through two transposed convolution operations. The other branch uses the output of the previous MLP as queries to perform token-to-image attention, generating two output streams: one is the IoU output token (1×256), which, after passing through an MLP layer, can predict the intersection over union (IoU) between each predicted mask and the ground truth mask; the other output is the output token for each mask (4×256), which, after passing through four MLP layers, results in a tensor. Finally, this tensor undergoes point-wise operations with the results from the first branch, iteratively computing the final predicted masks, which are the target products of the segmentation task.

When processing patients’ CT imaging data, we selected the slice with the largest cross-sectional area of the rectum. This selection strategy aims to obtain the image slice with the richest rectal region information, ensuring the accuracy and comprehensiveness of feature extraction. By processing these high-information-content slices, the SAM-Med2D model can fully utilize its strengths to extract significant imaging features related to radiation proctitis.

### Extraction of features in the rectal region

The image data of the patient is based on the slice with the largest cross-sectional area of the rectum. This slice image is used as one of the inputs to the SAM-Med2D model. Using the segmentation results provided by the hospital, a prompt point in the rectal region is identified. If a point has five neighboring pixels that also belong to the rectal region, it is considered a prompt point for the rectal area. This prompt point is input into the SAM-Med2D model to guide the model’s attention towards the rectal region and extract highly relevant features. As illustrated in Fig. 7, the segmentation results of the SAM-Med2D model closely match the hospital’s segmentation results, suggesting that the SAM-Med2D model effectively focuses on the rectal region.

We utilize the image embedding features obtained from the mask decoder as the features of the patient’s CT image, specifically located as shown in Fig. 4. These features are $16 \times 16$ in size with 256 dimensions, meaning that the image is divided into $16 \times 16$ depth feature patches. The features of the patch where the prompt point is located are used as the features of the rectal region for the patient, with a feature size of 256 dimensions. The calculation method for the patch coordinates is as follows:

8 $$\begin{aligned} (w\_index,h\_index) = (\left[ \frac{x}{image\_width}\right] \times 16,\left[ \frac{y}{image\_height}\right] \times 16), \end{aligned}$$

Here, $w\_index$ and $h\_index$ are the indices of the patch, $[\cdot ]$ denotes the floor function, $x$ and $y$ are the coordinates of the prompt point, and $image\_width$ and $image\_height$ are the width and height of the image, respectively.

### Feature selection

In the feature selection process, we employed the Standardized Mean Difference (SMD) and LASSO regression (Least Absolute Shrinkage and Selection Operator) methods. First, we classified the samples into two groups based on whether they had radiation proctitis. For each feature, we calculated the SMD between these two groups. Features with an SMD value greater than 0.2 were selected. Subsequently, we used the LASSO regression algorithm for further feature selection. LASSO is an embedded feature selection method that applies L1 regularization to shrink the coefficients of unimportant features to zero, thereby achieving feature selection. LASSO not only reduces the complexity of the model but also enhances its interpretability and predictive performance.

### Machine learning-based predictive model

After obtaining the significant features, we used these features to build a machine learning model to predict radiation proctitis. We selected three classic machine learning algorithms for modeling: Logistic Regression^32^, Random Forest^33^, and Gaussian Naive Bayes. Each method has its unique characteristics, allowing for analysis and prediction from different perspectives.

Logistic Regression is a linear model widely used for classification tasks. It estimates feature coefficients by maximizing the log-likelihood function to predict whether a patient will develop radiation proctitis. Its advantages include simplicity, high computational efficiency, and the ability to output the significance level of features.

Random Forest is an ensemble learning method based on decision trees. It improves the predictive performance and robustness of the model by constructing multiple decision trees and averaging their results. Random Forest can handle high-dimensional data and model complex nonlinear relationships between features effectively.

Gaussian Naive Bayes is a probabilistic classification method based on Bayes’ theorem, assuming that the features are independent and follow a Gaussian distribution. Although this assumption is quite strict in practice, Gaussian Naive Bayes has shown good classification performance in many applications, especially when the sample size is small.

## Results

### Experimental setup

**Fig. 5:**
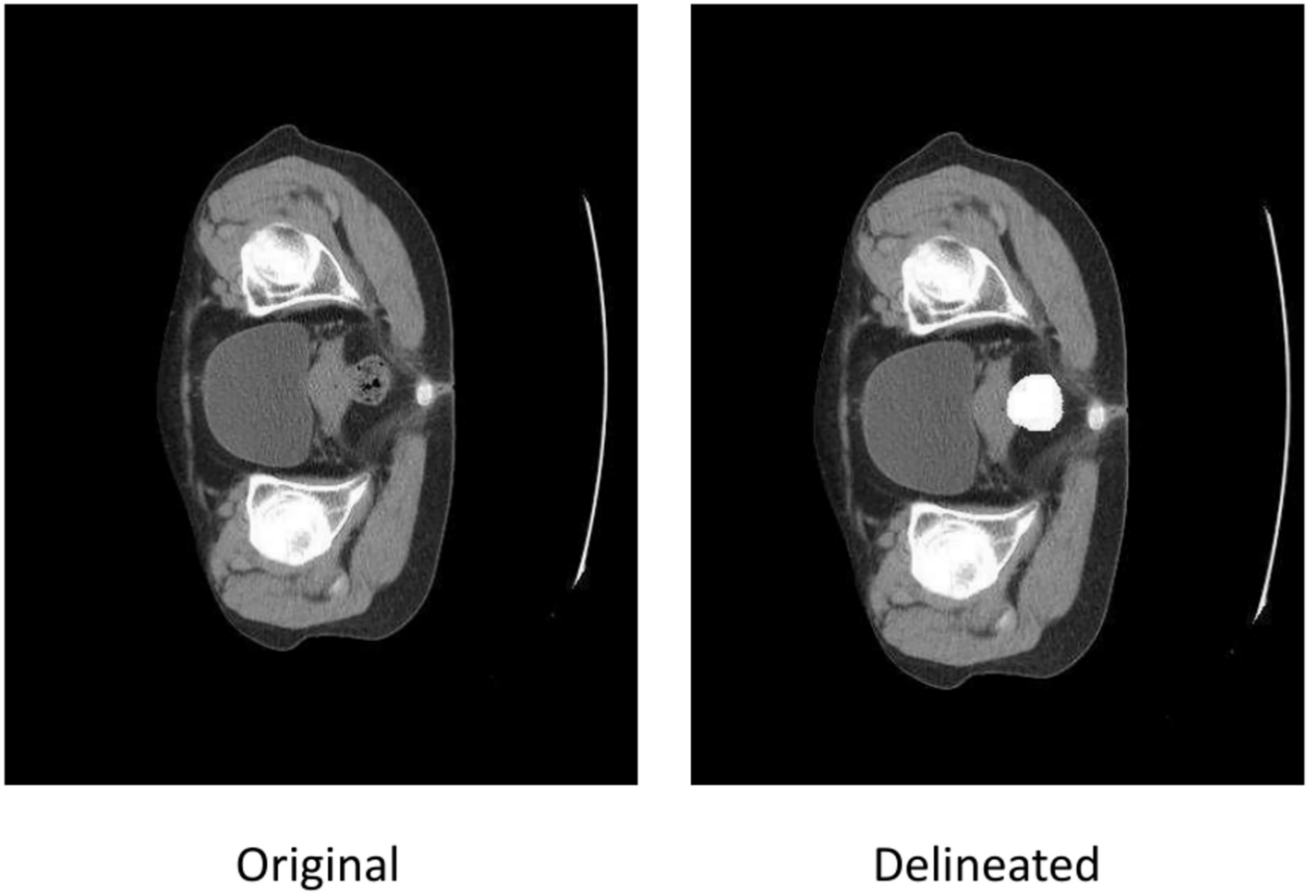
Overview of preprocessed CT data slices: the left image shows the patient’s CT data, while the right image highlights the rectal region delineated for that patient.

As shown in Fig. 5, professional physicians have delineated the rectal regions in these CT images. The task is to predict whether these patients will develop radiation proctitis after receiving radiation therapy. After treatment, 41 patients did not develop proctitis, while 109 did. The CT data for these 150 patients has a shape of 512X512XD, where D is greater than or equal to 50. For each patient’s CT image data, the slice with the largest cross-sectional area of the rectum is selected for feature extraction, as shown in Fig. 2. The experiments were conducted using Python version 3.8 and the deep learning framework PyTorch version 1.12.0.

### Evaluation metrics

This section details the commonly used performance evaluation metrics applied in this study:

*Accuracy*: This metric indicates the proportion of correctly predicted labels in the prediction results. Its advantage lies in its simplicity and straightforward interpretation. *Precision*: This metric represents the proportion of true positives among the samples predicted as true. It indicates how many of the predicted positive cases are actually positive. *Recall*: Also known as sensitivity, this metric measures the proportion of true positives correctly identified by the model. It indicates how many of the actual positive cases were captured by the model. *F1 Score*: This is the weighted harmonic mean of precision and recall, designed to avoid extreme values. It provides a balance between precision and recall. *AUC (Area Under the ROC Curve)*: This metric reflects the classifier’s ability to rank samples and is represented by the area under the ROC curve. The AUC value ranges from [0, 1], and it is particularly useful for datasets with imbalanced class distributions. These metrics collectively provide a comprehensive evaluation of the model’s performance, especially in handling class imbalance and ensuring both precision and recall are considered.

### Performance comparison

**Table 1:** Performance comparison of different classification models.

Model	Accuracy (95% CI)	Precision (95% CI)	Recall (95% CI)	F1 score (95% CI)	AUC (95% CI)
Logistic regression	0.77 (0.70 to 0.86)	0.78 (0.67 to 0.91)	0.94 (0.84 to 1.00)	0.85 (0.80 to 0.92)	0.73 (0.67 to 0.83)
Random forest	0.73 (0.61 to 0.89)	0.73 (0.61 to 0.88)	0.98 (0.95 to 1.00)	0.84 (0.76 to 0.94)	0.69 (0.61 to 0.80)
GuassianNB	0.69 (0.64 to 0.79)	0.75 (0.66 to 0.87)	0.84 (0.76 to 0.90)	0.80 (0.74 to 0.87)	0.63 (0.48 to 0.75)

As show in Table 1, the experimental results demonstrate that the features extracted by the SAM-Med2D model, which is based on Transformer architecture, are highly effective in predicting radiation proctitis. The high recall rates across all models suggest that the SAM-Med2D model is proficient at identifying positive cases of radiation proctitis. This is critical in medical diagnosis, where missing a positive case can have serious consequences.

**Fig. 6:**
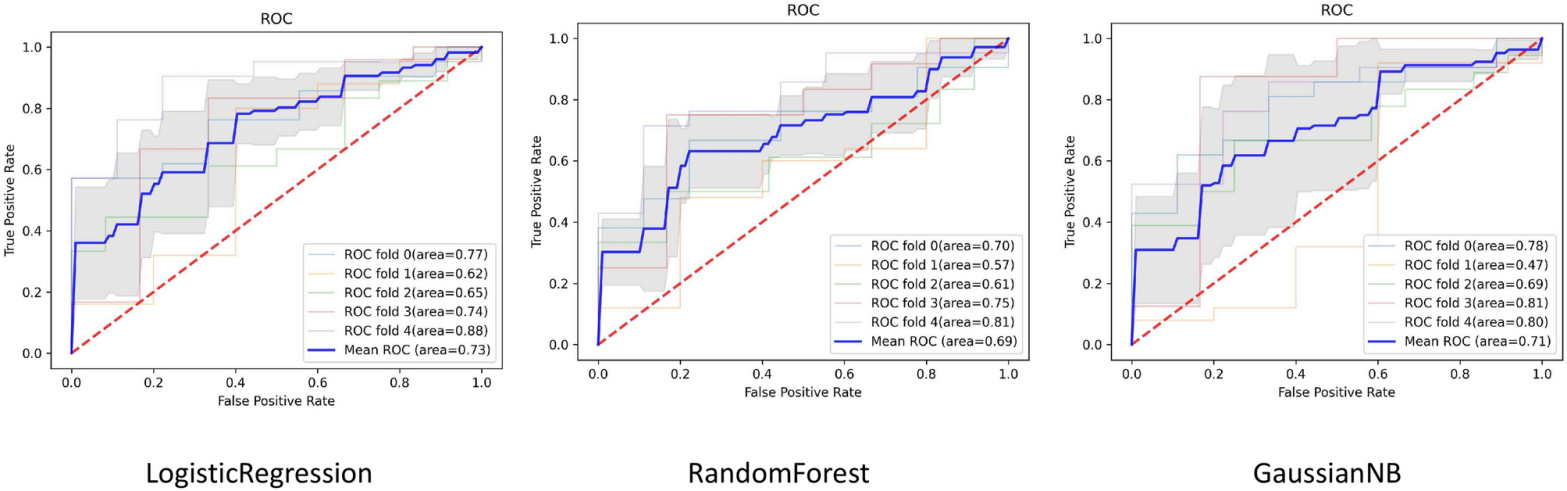
The ROC curves for three classification models: Logistic Regression, Random Forest, and Gaussian Naive Bayes, with each plot showing the performance across different folds and the mean ROC curve..

As show in the Fig. 6, it presents the ROC curves for the Logistic Regression, Random Forest, and Gaussian Naive Bayes methods, utilizing a 5-fold cross-validation approach. The gray area represents the confidence interval for the mean ROC curve. Notably, the confidence intervals for the ROC curves of Logistic Regression and Random Forest are smaller than Gaussian Naive Bayes. Among the methods, Logistic Regression demonstrates the best performance, achieving an AUC value of 0.73, while Random Forest ranks second with an AUC of 0.69 and the GaussianNB third with an AUC value of 0.63.

### Visualization

**Fig. 7:**
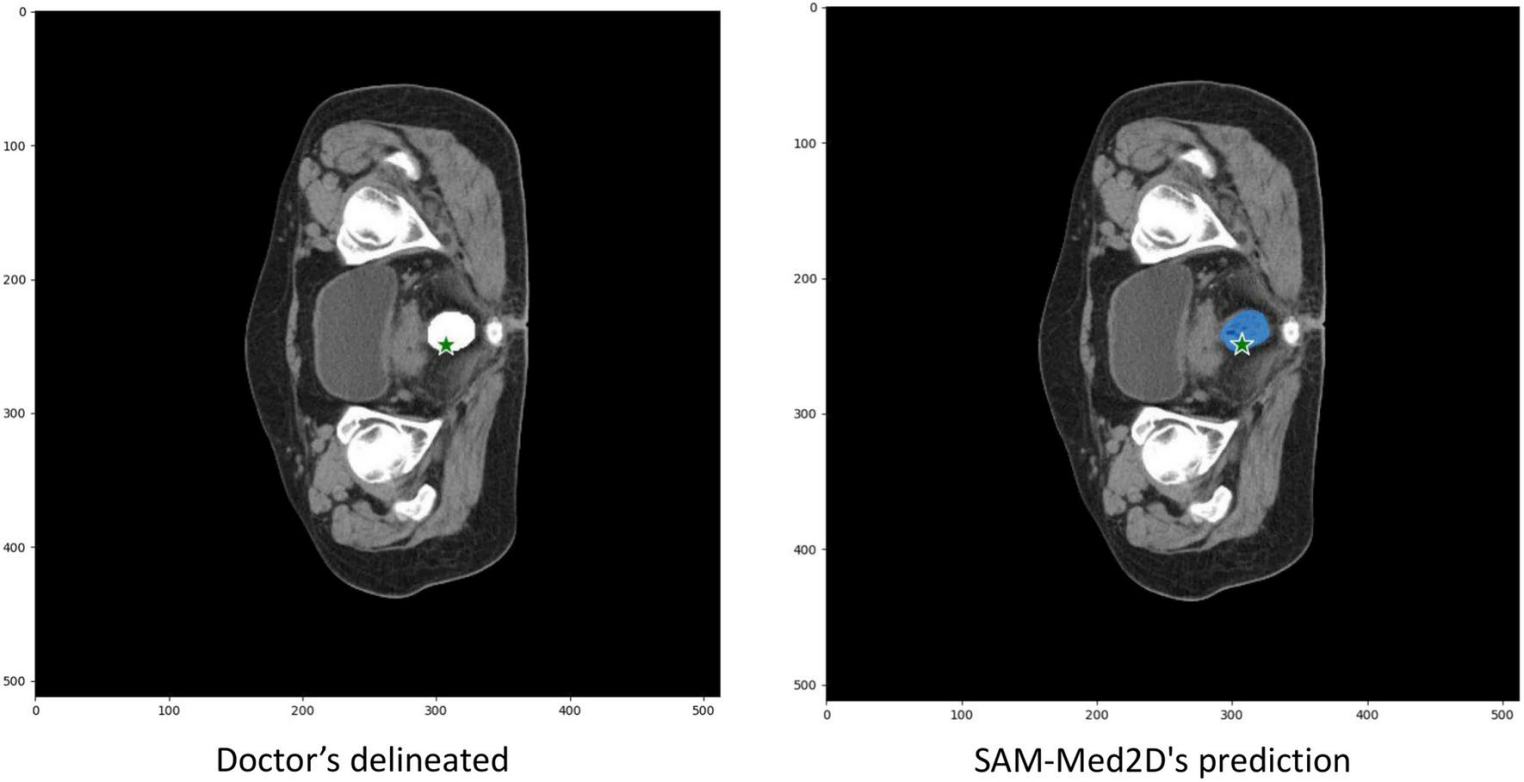
Performance of the SAM-Med2D model: the left image shows the doctor’s delineated results, with green asterisks indicating the prompt points used, and the right image displays the SAM-Med2D’s prediction in blue.

The slice image with the largest cross-sectional area of the rectum is used as the patient’s image data and serves as one of the inputs to the SAM-Med2D model. Based on the segmentation results provided by the hospital, a prompt point in the rectal region is identified. If a point has five neighboring pixels that also belong to the rectal region, it is considered a prompt point for the rectal area. This prompt point is then input into the SAM-Med2D model to guide the model’s attention towards the rectal region and extract highly relevant features. As shown in Fig. 7, the segmentation results of the SAM-Med2D model closely match those provided by the hospital, indicating that the SAM-Med2D model effectively focuses on the rectal region.

## Discussion

This study explores the importance of features extracted by the Transformer-based SAM-Med2D model for predicting radiation proctitis. The predictive models built using features extracted by the SAM-Med2D model achieved AUC values around 0.73, demonstrating good performance. Compared to some classical deep learning models (such as ResNet and CNN) and other Transformer-based feature extraction models (such as CLIP/ViT and SwinUnet), SAM-Med2D exhibits superior feature extraction capabilities for medical images. The segmentation results indicate that the SAM-Med2D model can effectively segment the rectal region, suggesting strong feature extraction abilities and an excellent focus on essential features. This implies that SAM-Med2D can obtain more accurate and representative medical image information, making the extracted features more effective for predicting radiation proctitis, and providing the prediction method with reasonable interpretability.

Traditional radiomics methods for clinical prognosis generally rely on manually designed features, such as post-radiation rectal wall thickness and the rate of change in rectal wall thickness before and after radiation. The workflow typically involves extracting predefined features from the region of interest. In contrast, the SAM-Med2D model, being a segmentation model itself, does not require the segmentation of regions of interest, which helps eliminate the segmentation step, improve efficiency, avoid errors, and allows for the automatic extraction of discriminative high-level features from low-level ones.

Different classification models, evaluation schemes, patient numbers, and label ratios can lead to varying prediction accuracies. The 5-fold cross-validation used in this study provides more stable and accurate evaluation, while T-tests and LASSO regression help reduce overfitting. However, one limitation of this study is the relatively small sample size, which may lead to unstable estimation accuracy and suboptimal model performance. More training samples could improve performance, and deep learning-based feature extraction methods can be applied to other imaging modalities, potentially further enhancing prediction accuracy.

The SAM-Med2D-based model is used to extract visual features from images, and deep learning-based methods are often considered black boxes. If the occurrence of radiation proctitis is associated with multiple factors, such as the dosage during radiation therapy, using only pre-radiation patient imaging data to predict radiation proctitis might not yield optimal results. To improve the performance of predictive models, it is often necessary to incorporate additional data to support and rationalize the prediction method. For example, considering visual imaging alone, data from patients immediately after radiation therapy, before the onset of radiation proctitis, could be included. By comparing visual features or changes in these features before and after radiation therapy, the prediction of radiation proctitis could be improved.

## Data Availability

The data used to support the findings of this study are available upon request from the corresponding authors.
